# Peer Victimization in Overweight Adolescents and Its Effect on Their Self-Esteem and Peer Difficulties

**DOI:** 10.3390/ijerph17010016

**Published:** 2019-12-18

**Authors:** David Álvarez-García, Andrea Núñez, María del Carmen Pérez-Fuentes, José Carlos Núñez

**Affiliations:** 1 Department of Psychology, University of Oviedo, Plaza Feijóo, s/n, 33003 Oviedo, Spain; jcarlosn@uniovi.es; 2 Department of Psychology, University of Almería. Ctra, Sacramento s/n, 04120 Almería, Spain; Uxxicom@ual.es (A.N.); mpf421@ual.es (M.d.C.P.-F.); 3 Faculty of Social Sciences and Humanities, Technical and Artistic University of Paraguay, Mayor Sebastián Bullo, s/n, Asunción 1628, Paraguay

**Keywords:** victimization, cybervictimization, overweight, adolescents, self-esteem, peer difficulties, shyness, social anxiety

## Abstract

This study has three objectives: to examine whether adolescents who perceive themselves as overweight differ from others in terms of offline victimization at school, cybervictimization, self-esteem, and difficulties relating to peers; to examine the possible effects of offline and cybervictimization on self-esteem and difficulties relating to peers; and to examine the possible moderating role of perceiving oneself as overweight on those effects. Previously validated questionnaires were applied to a sample of 3145 adolescents in Asturias (Spain). Descriptive, inferential, correlational, and structural equation analyses were performed. Adolescents who perceived themselves as overweight reported being victims of both offline victimization and most forms of cybervictimization to a greater extent than those who did not perceive themselves as overweight. They also reported lower self-esteem and more peer difficulties (shyness or social anxiety). In both groups of adolescents, victimization and cybervictimization were correlated with each other, both types of victimization had direct, negative effects on self-esteem, and self-esteem in turn had a direct, negative effect on peer difficulties. Furthermore, offline victimization had a direct, positive effect on peer difficulties. Perceiving oneself as overweight moderated the effect of self-esteem on peer difficulties. In adolescents perceiving themselves as overweight, low self-esteem was a stronger risk factor of peer difficulties than in the rest of the adolescents. With high overall self-esteem there were no significant differences in peer difficulties between the adolescents perceiving themselves as overweight and the rest of the adolescents.

## 1. Introduction

Adolescence is a critical stage in the formation of personal identity, during which there are significant physical, psychological and social changes [1]. Increases in hormone activity lead tosexual organs maturing and the development of secondary sexual characteristics, such as changes in the body’s composition and the tone of voice, the appearance of thicker body hair, or the appearance of acne. In adolescence the ability to posit hypotheses is consolidated, as is the ability to think about what is possible or not about current reality. Adolescents become more aware of the possibility of other worlds, other models of the family, other lives, and other bodies, for instance, which can cause insecurity or dissatisfaction. During adolescence, children become increasingly independent of their parents. Their main references and confidants are their friends, and interest in romantic relationships appears.

Early friendship groups are often forged around conversations in which physical appearance or physical ability in shared interests are very important. Appearance and physical ability are therefore very important for acceptance and status in the group [2,3]. For adolescents, peer acceptance is extremely important, so much so that it is likely that peers’ attitudes and judgements will affect self-concept and self-esteem, and in turn, how adolescents relate to their peers in future.

In this context, being overweight can be a significant problem during adolescence. In fact, the World Health Organization highlights being overweight in childhood and adolescence as one of the main current challenges for public health, for two main reasons. One is that there is a worldwide increasing trend in its prevalence [4]. It is estimated that in Spain at the present time, more than a third (34.9%) of children and adolescents aged between 8 and 16 are overweight or obese [5]. In addition, being overweight can have very negative consequences for health and wellbeing. Physically, it increases the chances of suffering from cardiovascular diseases, asthma, type II diabetes, osteoarthritis, chronic back pain, gallbladder disease, and some types of cancer [6]. Psychologically, being overweight may affect relationships with peers and psychosocial wellbeing [7]. Being overweight is one of the main reasons for rejection and being the victim of aggression in school environments [8,9]. Both boys and girls who are overweight experience significantly more bullying than adolescents who are a normal weight [10].

Some research about this problem has shown that overweight adolescents are more likely to suffer from cybervictimization than other types of victimization [11]. Although it has been little studied, it is possible that the anonymity and distance encourage disinhibited behavior by the aggressor, and make it harder for them to feel empathy for the suffering of their victim, which makes it more likely for the aggression to occur and be repeated [12]. Nonetheless there are many studies suggesting that while offline- and cyber-victimization differ in their details and may not occur together, they are highly correlated [13,14].

Previous studies have highlighted that self-perceptions of being overweight were more strongly associated with the experience of being bullied than objectively being overweight [15,16]. Dissatisfaction with body image seems to be a risk factor for falling victim to peer aggression, perhaps due to its negative effects on overall self-esteem and concomitant effects on difficulties of social interaction and isolation, which can increase the likelihood of being a victim. In general, how an adolescent is treated by people who are significant to them affects their self-esteem, and this in turn affects how they relate to others in the future. Peer victimization is negatively associated with self-esteem [17,18,19], and self-esteem is negatively correlated with shyness and social anxiety in adolescents [20,21,22,23].

To date, few studies have examined the modulating role of perceived overweight in the effect that being a victim of peer violence has on self-esteem and future difficulties relating to peers. This is despite the fact that children who were overweight exhibited significantly lower self-esteem than normal-weight children [24], and that low self-esteem—particularly body-esteem—increases the likelihood of experiencing social anxiety [23,25]. An adolescent’s perception of being overweight can be fed by the treatment they receive from their peers. Appearance and physical competence are usually one of the main focuses of aggressors’ criticism and comments; and negative comments about the body are a fundamental aspect of adolescents developing negative body images [26]. Both traditional victimization and cybervictimization are related with low body-esteem, although some studies have found that cybervictimization has a greater negative impact on body-esteem than traditional victimization [27]. In general, adolescents with low self-esteem try to avoid situations of social interaction or they face them nervously and reluctantly. Some studies indicate that body-esteem is particularly important in the relationship between self-esteem and social anxiety [23,25].

**Figure 1:**
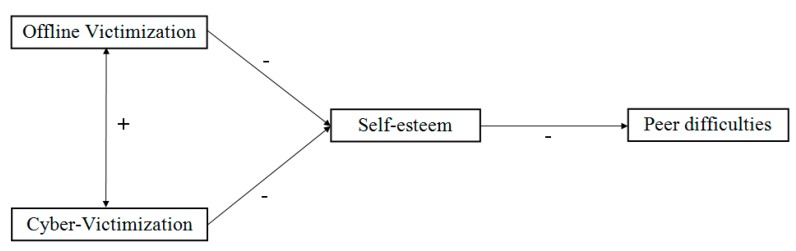
Starting theoretical model (+ = Positive relationship; − = Negative relationship).

Therefore, our study has the three following objectives. Objective 1—Analyzing whether those who perceive themselves as overweight differ from the others in terms of offline victimization, cybervictimization, self-esteem and difficulties relating to peers. We expect that students who perceive themselves as overweight will report more offline victimization and cybervictimization, lower self-esteem and greater difficulties in social interaction. Objective 2—Examining the association of offline victimization and cybervictimization with self-esteem and future difficulties in relationships, both in overweight students and normal-weight students. We expect offline and cybervictimization to be significantly related to each other, and both kinds of victimization to be negatively related to self-esteem, which in turn we expect to be negatively related to difficulties in future relationships (Figure 1). Objective 3—Analyzing the moderating role of perceiving oneself as overweight on the relationships found in the model. We expect the relationship between each of the variables to be stronger in adolescents who perceive themselves to be overweight compared to the others.

## 2. Materials and Methods

### 2.1. Participants

We analyzed the responses of 3145 adolescents in Asturias (Spain). The sample was almost evenly split between boys (50.7%) and girls (49.3%), aged between 12 and 18 years old (*M* = 14.03, SD = 1.40). Almost all of them (95.5%) had their own mobile phone. Most (83.9%) used the internet in their free time for non-homework activities. 93.3% used instant messaging applications (e.g., WhatsApp), and 78.2% participated in social networks (e.g., Facebook) in their free time.

The adolescents were from 19 different schools, selected via stratified random sampling from all of the state-funded schools in Asturias that provide compulsory secondary education. State-funded secondary schools make up 95.9% of the secondary school in Asturias. To select the sample, the population of schools was divided according to type (public or concerted) and the number in each strata was set in proportion to the number of schools in the population. In Spain, public schools are financed and run entirely by the state, whereas concerted schools are privately run but financed at least in part by public money. As a result, 11 public schools and 8 concerted schools were selected. The schools were predominantly in urban and socioeconomically middle-class areas. In each school, all of the students in the four years of compulsory secondary education (ESO) were evaluated.

In the sample as a whole, 122 adolescents (3.9% of the total) considered themselves to be among the chubbiest students in the school (used item and criteria are explained in section “2.2. Measuring Instruments”). There were no statistically significant differences between these adolescents and the others in any of the variables used to describe the sample (gender, age, having their own mobile phone, using the internet in their free time for non-homework activities, using instant messaging, and participating in social networks in their free time).

There were 3023 adolescents (96.1% of the total) who did not consider themselves to be among the chubbiest students in the school. Given this disparity in the size of the two sub-samples (perceived overweight and perceived normal-weight), and in order not to distort the calculations of both the correlations or regression coefficients and the fit of the model, a random sample of approximately 10% of the adolescents who perceived themselves as being a normal-weight was selected. This selected sample (*n* = 293) did not statistically significantly differ from the total sample of non-overweight adolescents (*n* = 3023) in any of the variables used to describe the sample, nor in any of the variables included in the starting theoretical model (Figure 1).

### 2.2. Measuring Instruments

#### 2.2.1. Sociodemographic data and use of communication technologies

We used an ad hoc questionnaire to collect information about subject age and gender, along with data on their use of mobile phones and the internet. They were asked about age in an open question. The remaining questions had dichotomous responses: boy/girl for gender; Yes/No for questions about mobile phones and the internet [“I have my own mobile phone”, “I use the internet in my free time for non-school tasks”, “In my free time I participate in social networks (Tuenti, Facebook or other)”, and “In my free time I use instant messaging services (Messenger, WhatsApp or other)”].

#### 2.2.2. Overweight 

To identify students who perceived themselves to be overweight, we used an item which asked them to give a qualitative evaluation of their weight. This is a similar strategy used by other authors in previous studies (e.g., [15,28,29]). The item we used was “I am among the chubbiest students in my school” [“Estoy entre los alumnos más rellenitos de mi Instituto”]. It used a Likert-type response with four alternatives (1 = Completely false; 2 = Somewhat false; 3 = Somewhat true; 4 = Completely true). In our study, we considered students to perceive themselves as overweight if they responded “Completely true”.

#### 2.2.3. Cybervictimization

We used the Cybervictimization Questionnaire for adolescents (CYVIC, [30]), to determine the frequency with which the subjects had been the subject of aggressions via mobile phone or the internet during the three months prior to the survey. It has 19 items and produces scores on four types of cybervictimization (impersonation, visual-sexual cybervictimization, verbal cybervictimization, and online exclusion) along with four additional indicators of visual cybervictimization related to teasing and happy slapping. The responses are in a Likert-type format (from 1 = Never, to 4 = Always). Scores for the four scales of each type of cybervictimization, as well as the overall scale, are calculated by adding together the scores of the items in the scales. High scores indicate high levels of cybervictimization. The internal consistency of the scale as a whole in our sample is moderate (α = 0.794; ω = 0.812; Greatest Lower Bound (GLB) = 0.859).

#### 2.2.4. Offline Victimization at School

To determine how often subjects suffered from aggressions in a physical context, in the school environment, we used the Offline School Victimization scale [31]. This is a self-report made up of six items: “Some of my classmates exclude me in games and activities at playtime”, “My classmates avoid me when we have to do group activities in class”, “My classmates make fun of me/laugh at me”, “My classmates say bad things about me behind my back”, “I have been insulted by a classmate to my face”, and “A student at the school has hit me, either in school or outside the school grounds”. The response is on a four-point Likert-type scale (1 = Never; 2 = Occasionally; 3 = Often; 4 = Always). The score in the overall scale is the sum of the item scores. High scores indicate high levels of offline victimization at school. The internal consistency of the scale with the sample in our study is moderate (α = 0.743; ω= 0.753; GLB= 0.799).

#### 2.2.5. Self-Esteem 

To determine how subjects evaluate themselves, we used a self-report scale with five items [31]: “I am happy with my physical appearance”, “I think I am a good person”, “I can do things at least as well as most of my classmates”, “I like how I am”, and “I feel proud of what I do”. The response is on a four-point Likert-type scale (1 = Completely false; 2 = Somewhat false; 3 = Somewhat true; 4 = Completely true). The score of the overall scale is the sum of the component item scores. High scores indicate high self-esteem. The internal consistency of the scale with the sample in our study is moderate (α= 0.737; ω= 0.748; GLB= 0.797).

#### 2.2.6. Peer difficulties

To determine the extent to which the subjects feel it difficult to relate to their peers we used a shyness-social anxiety scale. This self-report scale is made up of five items about inhibition and feeling uncomfortable relating to others, especially people who are not close friends [31]: “I’m shy and not very talkative, except with my friends”, “I often feel embarrassed saying hello to people”, “I get nervous when I have to be with a group of children that I don’t know well”, “I get uptight if I meet an acquaintance on the street”, and “I find it difficult to get to know new people, to make friends, or to start talking to people that I don’t know”. The response is on a four-point Likert-type scale (1 = Completely false; 2 = Somewhat false; 3 = Somewhat true; 4 = Completely true). The score of the overall scale is the sum of the component item scores. High scores indicate high levels of shyness/social anxiety (peer difficulties). The internal consistency of the scale with the sample in our study is moderate (α = 0.745; ω = 0.746; GLB = 0.779).

### 2.3. Procedure

Once the sample and the measuring instruments had been selected, permission was sought from the administration in each center to apply the questionnaires. The management of each school were informed of the study objectives and procedures, its voluntary, anonymous nature, and the confidential treatment of any results. Once schools agreed to participate, parents’ or guardians’ informed consent was sought as the subjects were minors. Before completing the questionnaires, the students were also assured that their participation was voluntary, confidential and anonymous. We asked them not to write their names on the questionnaire. We informed them that no one could know what they had answered and that their responses would be combined with responses from the other students from other schools in order to give general results and conclusions. In general, the students spent 20 min completing the questionnaires, although this was flexible depending on the age and the students themselves. The questionnaires were applied by the research team, during classroom hours.

### 2.4. Data Analysis

Firstly, we carried out descriptive analyses of the levels of victimization, cybervictimization, self-esteem and difficulties relating to peers in the two samples; perceived overweight and non-overweight (Objective 1). The possible differences between these two groups were examined via ANOVAs. Cohen’s *d* [32] was used as a measure of effect size, following this author’s criteria: *d* < 0.20, very small effect size; *d* between 0.20 and 0.50, small effect size; *d* between 0.50 and 0.80, moderate effect size; and *d* ≥ 0.80, large effect size.

Following that, we analyzed the association of victimization and cybervictimization with self-esteem and peer difficulties in both samples; overweight and not (Objective 2). To do that, we first calculated the correlation coefficients between the four variables. Then we performed a path analysis with each sample to look at the fit of the starting theoretical model to the data, and the size of the effects between the variables. The fit of the model was evaluated using the chi-squared statistic (*χ*^2^), the chi-squared/degrees of freedom ratio (*χ*^2^/df), the adjusted goodness-of-fit index (AGFI), the comparative fit index (CFI), and the root mean square error of approximation (RMSEA). Fit is considered acceptable when *χ*^2^ has a *p* > 0.05, *χ*^2^/df < 3, AGFI ≥ 0.95, CFI ≥ 0.95 and RMSEA < 0.08 [33].

Finally, we examined the moderating role of the perceived overweight on the statistically significant effects from the previous phase (Objective 3). To do that, we analyzed the interaction effect between the moderating variable (perceived overweight or not) and the independent variable in each pair of related variables in the model. Following that we analyzed the possible impact of the level of self-esteem on the differences in peer difficulties in overweight and non-overweight adolescents.

For the descriptive analyses, inferential analyses, and simple correlations we used SPSS 22.0 (IBM, Armonk, NY, USA) [34]. For the path analysis we used AMOS 22.0 (IBM, Chicago, IL, USA) [35], and to look at whether perceived overweight could moderate each of the statistically significant relationships we used the PROCESS [36] module of the SPSS statistical package.

## 3. Results

*Objective 1—Differences in offline victimization, cybervictimization, self-esteem, and peer difficulties between adolescents who perceive themselves to be overweight and those who do not.*

Table 1 shows the descriptive data, and the differences in victimization, cybervictimization, self-esteem, and difficulties relating to peers between adolescents who perceive themselves to be overweight (*n* = 122) and those who do not (*n* = 3023).

The results indicate that those adolescents who perceive themselves to be overweight reported being victims of both offline victimization at school and some types of cybervictimization more than adolescents who did not perceive themselves to be overweight.

Regarding the offline victimization at school, adolescents who perceive themselves to be overweight score higher in this factor than the rest of the adolescents. Item by item analysis showed that, in all of the items (or types of offline victimization at school), the adolescents who perceive themselves to be overweight reported suffering more violence than the others. The differences were statistically significant in all items (*p* ≤ 0.001), with small or very small effect sizes (Cohen’s *d* between 0.16 and 0.23). This item by item analysis is not included in Table 1 for reasons of concision.

In terms of cybervictimization, adolescents who perceived themselves to be overweight scored more highly in this factor than the others. However, not all of the types of cybervictimization produced statistically significant differences. Perceived overweight adolescents reported being victims of impersonation, written-verbal cybervictimization and online exclusion to a greater extent than the others. In contrast, there were no statistically significant differences in most of the visual types of cybervictimization with sexual connotations, nor in most cybervictimization behaviors consisting of teasing or happy slapping. The only visual cybervictimization in which there were statistically significant differences was being the victim of having compromising photos or videos of them posted on the Internet without their permission, to harm them or make fun of them, which was more common in adolescents perceiving themselves as overweight. The magnitude of the differences was generally small or very small. The effect sizes were larger in offline victimization at school than cybervictimization.

Table 1 also shows the differences between adolescents who perceive themselves to be overweight and those who do not in the possible effects of peer aggression. Overweight adolescents reported having lower self-esteem, and more peer difficulties (shyness or social anxiety) than the other adolescents. The size of the differences was small for empathy, and very small for peer difficulties.

*Objective 2—The relationship of offline victimization and cybervictimization with self-esteem and peer difficulties in adolescents perceiving themselves as overweight and adolescents who do not perceive themselves as overweight.*

Table 2 shows the correlation coefficients between the variables in the theoretical starting model (Figure 1), from the sample of perceived overweight adolescents (*n* = 122) and the reduced sample of not perceived overweight adolescents (*n* = 293). All of the correlations were statistically significant, except for the correlation between cybervictimization and peer difficulties in the not perceived overweight sample. In both samples this relationship (cybervictimization—peer difficulties) had the lowest correlation coefficients.

**Figure 2:**
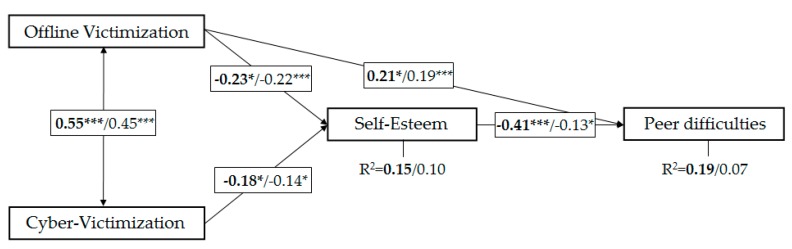
Path model. All coefficients are standardized. On the left, in bold, results from the sample of adolescents perceiving themselves to be overweight (*n*=122); on the right, results from the sample of adolescents who do not perceive themselves as overweight (*n*=293). * *p* ≤ 0.05; *** *p* ≤ 0.001.

Data relating to the fit of the starting path model (Figure 1) in both samples indicated that, although some of the indexes suggested an acceptable fit, most advised modifying the model for both samples. The modification indices and the expected change in chi-squared demonstrated the advisability of re-specifying the starting model, adding the direct effect of offline victimization at school on peer difficulties (Figure 2). Given the statistical and theoretical sense this made, the fit of the re-specified model was examined for both samples: students perceiving themselves to be overweight [*χ*^2^ = 0.072; *df* = 1; *p* = 0.789; *χ*^2^/*df* = 0.072; AGFI = 0.997; CFI = 1.000; RMSEA = 0.000 (90% CI 0.000–0.156); Akaike Information Criterion (AIC) = 18.072]; and students not perceiving themselves to be overweight [*χ*^2^ = 0.841; *df* = 1; *p* = 0.359; *χ*^2^/*df* = 0.841; AGFI = 0.986; CFI = 1.000; RMSEA = 0.000 (90% CI 0.000–0.150); AIC = 18.841]. In addition the difference in *χ*^2^, along with the values of AIC suggested that the model fit in both cases was similarly good (Δ*χ*^2^ = 0.769; *p* > 0.05).

Table 3 gives the results for both samples of adolescents of the relationships found between the variables making up the model.

The data in Table 3 confirm the hypothesized relationships in the starting model (Figure 1): in both samples offline victimization and cybervictimization were positively correlated with each other; both types of victimization were negatively correlated with self-esteem; and self-esteem was in turn negatively correlated with peer difficulties. Furthermore, we found offline victimization to be directly associated with peer difficulties. In general, effect sizes were small, except for the effect of self-esteem on peer difficulties in the sample of adolescents perceiving themselves to be overweight, where it was moderate to large; and the size of the correlation between the two types of victimization (offline and cyber), which was large in both samples.

The indirect effects of both offline victimization and cybervictimization on peer difficulties were not statistically significant in either of the two samples [adolescents who do not perceive themselves as overweight (Cibervictimization→Peer Difficulties = 0.018; Offline Victimization→ Peer Difficulties = 0.027), and adolescents perceiving themselves to be overweight (Cibervictimization→Peer Difficulties = 0.069; Offline Victimization→ Peer Difficulties = 0.080)]. The percentage of variance explained in self-esteem and peer difficulties was low in both samples, particularly in adolescents who do not perceive themselves as overweight: non-overweight sample (R^2^_SE_ = 0.095; R^2^_PD_ = 0.067), overweight sample (R^2^_SE_ = 0.146; R^2^_PD_ = 0.193). Figure 2 shows the most important results.

*Objective 3—The moderating role of perceiving oneself as overweight on the identified relationships.*

We examined the possible moderating role of perceiving oneself as overweight on each of the relationships that were found to be significant in the fit of the path model. As Table 4 indicates, perceiving oneself as overweight does not moderate the relationship between the variables in the model, with the exception of the relationship between self-esteem and peer difficulties, in which the moderating effect is marginally significant.

**Figure 3:**
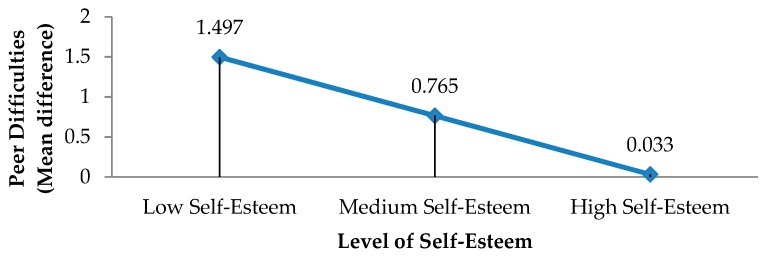
Differences in peer difficulties between adolescents perceiving themselves to be overweight and adolescents who do not perceive themselves as overweight, according to level of self-esteem.

A more detailed analysis of the moderating role of perceiving oneself as overweight on the relationship between self-esteem and peer difficulties (Table 5) shows that it depends on the level of self-esteem. The differences in peer difficulties between adolescents perceiving themselves to be overweight and adolescents who do not perceive themselves as overweight are substantial when self-esteem is low and disappear when self-esteem is high. With low self-esteem, adolescents perceiving themselves to be overweight have substantially higher levels of peer difficulties than adolescents perceiving themselves not to be overweight. As levels of self-esteem increase, the differences between the two groups shrinks, until it disappears when self-esteem is high (Figure 3): low self-esteem (mean difference = 1.497), moderate self-esteem (mean difference = 0.765), high self-esteem (mean difference = 0.033).

## 4. Discussion

The first objective of this work was to examine whether, in a sample of Spanish adolescents, those who perceive themselves to be overweight differed from the others in offline victimization, cybervictimization, self-esteem, and peer difficulties (Objective 1). The results partially support the starting hypothesis: students perceiving themselves as overweight reported, as expected, more offline victimization at school [8,9,10], lower self- esteem [24], and greater difficulties in social interaction [25]. However, not in all types of cybervictimization examined were there statistically significant differences.

Previous research has shown that adolescents perceiving themselves as overweight usually report more cybervictimization in general than other adolescents [37]. However, to date the possible differences depending on the type of cybervictimization have hardly been studied. In this study, adolescents perceiving themselves as overweight, as expected, reported higher overall scores for cybervictimization. However, when the different types of cybervictimization are examined, perceived overweight adolescents report more impersonation, written-verbal cybervictimization, and online exclusion, but do not report more victimization in most of the visual types of cybervictimization included in this study.

We did not find differences in visual cybervictimization with a sexual connotation. One possible reason for this may be that the greater tendency perceived overweight adolescents have to suffer cybervictimization may compensate for the lower likelihood that they engage in sexting, found in previous research [38]. Two of the three items making up the visual cybervictimization with a sexual connotation factor, refer to aggressions in which this risky behavior is involved. Another possible reason may be that when real photos or videos of overweight people are taken or shared, it is usually done to make fun of them, rather than having suggestive or sexual connotations. The third of the three items of the visual cybervictimization with sexual connotations factor, which did not exhibit differences depending on perceptions of being overweight, asks if someone has taken without permission and disseminated images of them with a sexual or suggestive content. In contrast, overweight adolescents do report more than the others that someone has posted real compromising photos or videos of them on the internet without their permission, to harm or make fun of them. This is the only indicator (item) referring to visual cybervictimization with a connotation of teasing or happy slapping in which there were differences. There were no differences in the two items around happy slapping (“I have been beaten, and others have recorded it and then disseminated it” and “They have forced me to do something humiliating, they have recorded it, and then disseminated it to ridicule me”) due to the low frequency in both overweight and non-overweight adolescents. As far as we are aware, no research has been published to date examining the possible differences in being a victim of happy slapping between self-perceived overweight adolescents and non self-perceived overweight adolescents.

Our second objective in this study was to look at the relationship of offline- and cybervictimization with self-esteem and difficulties relating to peers in both groups of students (Objective 2). Our results support the hypothesized relationships in the theoretical starting model (Figure 1). Firstly, offline victimization and cybervictimization were significantly and positively related to each other. This is in line with previous research noting offline victimization and cybervictimization as two distinct, but related, constructs [39,40]. Secondly, both offline victimization and cybervictimization were negatively related to self-esteem. This is also in line with prior research [18,19]. Thirdly, self-esteem was negatively related to difficulties relating to peers, in the form of shyness and social anxiety. This is also in agreement with previous research [20,21,22,23].

Our results also demonstrated an effect which was not considered in the theoretical starting model. Offline victimization had an effect on peer difficulties, which is not only indirect via self-esteem, but also direct. In contrast, the direct effect of cybervictimization on peer difficulties was not statistically significant in our study. Previous research has concluded that traditional victimization, principally relational, has more impact on social anxiety than cybervictimization [41]. One possible explanation is that shyness/social anxiety, as evaluated in our study, and as evaluated traditionally, takes place in face-to-face situations. It is possible that bad experiences in face-to-face social relations have greater negative impact in the future on exactly these types of relationships than negative experiences via electronic devices.

Our third and final objective in this study was to examine the moderating role of self-perceived overweight on the relationships we had identified (Objective 3). Despite this being something that had been seldomstudied explicitly in previous research, the literature review suggested that perceiving oneself overweight could accentuate the negative effects of offline and online victimization on self-esteem, as well as the negative effect of self-esteem on social anxiety [23,25]. Our results show that while the intensity of the effects was greater in the sample of self-perceived overweight adolescents in all of the relationships examined, perceptions of being overweight only had a significant moderating effect on the relationship between self-esteem and peer difficulties. More precisely, adolescents with low self-esteem exhibited more peer difficulties than others, and within adolescents with low self-esteem, those who perceive themselves to be overweight exhibited substantially more peer difficulties than those who do not perceive themselves to be overweight. In contrast, adolescents with high self-esteem exhibited fewer peer difficulties than the rest, and in high self-esteem adolescents there were no differences found in peer difficulties between adolescents perceiving themselves as overweight and those perceiving themselves as not overweight.

One possible explanation is that in adolescence, appearance and physical ability are fundamental to being part of peer groups. Friendship groups in adolescents are usually built around shared interests, in which physical skill is important, or around conversations in which body image is evaluated or criticized. Having low self-esteem in a physical sense may contribute to being reserved when it comes to establishing friendships, or maintaining relationships with people who are not yet close. However, adolescents who make up for this negative physical self-concept with higher self-concepts in other areas may have fewer peer difficulties.

This study is a contribution to the analysis of the effects of peer victimization and being overweight in adolescence, and has significant theoretical and practical implications. From a theoretical point of view, our study contributes to the understanding of the negative consequences of offline victimization and cybervictimization on the victim’s self-esteem and peer difficulties, and the moderating role of perceptions of being overweight on these consequences. From a practical perspective, the present study highlights the importance of preventing aggressions and cyber-aggressions between adolescents, due to their negative impact on young people’s psychosocial wellbeing. This study also highlights the importance of encouraging positive attitudes to diversity in general, and overweight people in particular. Encouraging a safe, respectful, mutually supportive school climate contributes to students’ psychosocial wellbeing, and through that, better learning and healthier development. Our study contributes to the identification of adolescents who are at particular risk of being victims of aggressions from their peers, with the aim of prevention or early detection. Our study advises not only the importance of encouraging a healthy diet and lifestyle for overweight people [42], but also the importance of strengthening their self-esteem [43] and working on attitudes and prejudices towards people who are overweight [44]. This must be a shared responsibility between family, school and society. Low self-esteem, which may be accentuated if one is the victim of peer aggression, may be a risk factor for eating disorders [24] and other mental health problems [45]. Cybervictimization has been associated with body dissatisfaction and overweight preoccupation [26,46]. Meta-analyses have indicated that avoidance of provocation or bullying is among the main motivators for weight loss [47], and that people with eating disorders were more likely to have been teased about their appearance or bullied prior to the onset of their eating disorder [48].

While this work is a contribution with significant theoretical and practical implications, it is important to recognize its limitations, which future studies should address. One limitation is that it is a cross-sectional study, which means that one cannot make any causal inferences. It would be useful to complement this analysis with longitudinal studies which would help to more accurately clarify the direction of the relationships between the variables. Another limitation is that in this study we only used one subjective measure of being overweight, and no objective measures. Although previous research has found a broad overlap between these two types of measure, self-reported overweight may not necessarily reflect actual overweight. In this study, perceived overweight seemed particularly important to us, in so far as the treatment and comments from peers may affect this perception. Nevertheless, future research should look at complementing this analysis by using objective measures of being overweight. Finally, a third limitation is that in this study we did not distinguish between being overweight and obesity. It would be useful for future studies to look at whether there are differences between overweight and obese adolescents in the results we found in this study.

## 5. Conclusions

Adolescents who perceive themselves to be overweight generally tend to be more likely to be victims of aggressions or cyber-aggressions from other adolescents, as well as being more likely to have lower self-esteem and more peer difficulties. The only exception we found was that there were no differences in visual cybervictimization with a sexual connotation, or in happy slapping-type visual cybervictimization.

Being a victim and being a cyber-victim of peer aggression are distinct phenomena that often appear together. Both victimization and cybervictimization are risk factors for low self-esteem, which in turn increases the likelihood of having peer difficulties (shyness and symptoms of social anxiety). Offline victimization has a greater impact on peer difficulties than cybervictimization.

Perceiving oneself as being overweight moderates the effect of general self-esteem on peer difficulties. In adolescents who perceive themselves to be overweight, low self-esteem is a more powerful risk factor for peer difficulties than in other adolescents. The differences in peer difficulties between adolescents perceiving themselves as overweight and those not doing so are substantial when general levels of self-esteem are low, but null when levels of self-esteem are high.

## Figures and Tables

**Table 1:** Differences in offline victimization, cybervictimization, self-esteem and peer difficulties between overweight and not overweight adolescents (*N* = 3145).

Variable	Theoretical Range		Overweight (*n* = 122)	Not Overweight (*n* = 3023)			
	Min.	Max.	M (SD)	M (SD)	F_(1,3143)_	*p*	*d*
Victimization							
Offline victimization at school	6	24	9.93 (3.75)	8.09 (2.43)	63.780	<0.001	0.29
Cybervictimization (Total)	19	76	22.89 (4.16)	21.60 (3.30)	17.524	<0.001	0.16
Impersonation	3	12	3.40 (0.93)	3.20 (0.62)	11.840	0.001	0.13
Written-Verbal	6	24	8.60 (2.61)	7.81 (2.10)	16.223	<0.001	0.14
Online exclusion	3	12	3.64 (1.31)	3.36 (0.79)	13.734	<0.001	0.13
Visual-Sexual	3	12	3.09 (0.32)	3.12 (0.47)	0.602	0.438	0.07
Visual-Teasing/Happy slapping							
Someone has posted doctored (modified) photos of me on the Internet to harm me or laugh at me.	1	4	1.06 (0.27)	1.04 (0.23)	0.378	0.539	0.06
Someone has posted real compromising photos or videos of me on the Internet without my permission, to harm me or make fun of me.	1	4	1.07 (0.26)	1.03 (0.20)	4.973	0.026	0.09
I have been hit, and others have recorded it and then disseminated the video.	1	4	1.02 (0.18)	1.01 (0.15)	0.017	0.897	0.01
They have forced me to do something humiliating, they have recorded it, and then disseminated it to ridicule me.	1	4	1.01 (0.09)	1.01 (0.14)	0.288	0.591	0.05
Effects							
Self-esteem	5	20	14.85 (3.67)	16.68 (2.77)	49.756	<0.001	0.26
Peer difficulties (shyness/social anxiety symptoms)	5	20	12.38 (4.35)	10.98 (3.70)	16.658	<0.001	0.14

**Table 2:** Pearson correlation coefficients for the sample of adolescents perceiving themselves to be overweight (*n* = 122) and the reduced sample of adolescents who do not perceive themselves as overweight (*n* = 293).

Variables	Offline Victimization at School	Cybervictimization	Self-Esteem	Peer Difficulties
Offline Victimization at School	--	0.551 **	−0.344 **	0.297 **
Cybervictimization	0.448 **	--	−0.330 **	0.230 *
Self-Esteem	−0.281 **	−0.241 **	--	−0.405 **
Peer Difficulties	0.229 **	0.071	−0.181 **	--

Note: Top right above diagonal, sample of adolescents perceiving themselves to be overweight (*n* = 122); botton left under diagonal, sample of adolescents perceiving themselves not as overweight (*n* = 293). ** *p* ≤ 0.01; * *p* ≤ 0.05.

**Table 3:** Fit of the path model in adolescents perceiving themselves to be overweight and adolescents perceiving themselves not to be overweight.

Direct Effects	*b*	s.e.	*t*	*p*	*d*
Adolescents who do not perceive themselves as overweight (*n* = 293)					
Offline Victimization → Self-Esteem	−0.216	0.069	−3.473	<0.001	0.41
Cybervictimization→ Self-Esteem	−0.144	0.046	−2.309	0.021	0.27
Self-Esteem → Peer Difficulties	−0.127	0.082	−2.157	0.031	0.25
Offline Victimization → Peer Difficulties	0.193	0.091	3.279	0.001	0.39
Offline Victimization ↔ Cybervictimization	0.448	0.622	6.982	<0.001	0.89
Adolescents perceiving themselves to be overweight (*n* = 122)					
Offline Victimization → Self-Esteem	−0.228	0.099	−2.316	0.021	0.43
Cybervictimization→ Self-Esteem	−0.177	0.089	−1.998	0.046	0.37
Self-Esteem → Peer Difficulties	−0.407	0.103	−3.951	<0.001	0.76
Offline Victimization → Peer Difficulties	0.208	0.101	2.059	0.040	0.38
Offline Victimization ↔ Cybervictimization	0.551	0.605	5.309	<0.001	1.09

**Table 4:** Effect of perceiving oneself as overweight.

Interaction Effects	Coefficient	SE	T	*p*
Outcome: Self-Esteem				
Int.:D1 x Offline Victimization	−0.025	0.099	−0.250	0.803
Int.: D_1_ xCybervictimization	−0.113	0.079	−1.423	0.156
Outcome: Peer Difficulties				
Int.: D_1_ x Offline Victimization	−0.009	0.132	−0.066	0.948
Int.: D_1_ x Self-Esteem	−0.229	0.126	−1.811	0.071

Note: Int. = Interaction; D_1_ = Perceiving oneself as overweight.

**Table 5:** Peer difficulties between adolescents perceiving themselves to be overweight and adolescents who do not perceive themselves as overweight, according to level of self-esteem.

Self-Esteem	Self-Perceived Overweight	Peer Difficulties
Low (12.937)	0	11.802
	1	13.299
Medium (16.140)	0	10.995
	1	11.760
High (19.344)	0	10.188
	1	10.221

Note: 0 = Not self-perceived overweight (*n* = 293); 1 = Self-perceived overweight (*n* = 122).
